# Association between *ADAM33* Single-Nucleotide Polymorphisms and Treatment Response to Inhaled Corticosteroids and a Long-Acting Beta-Agonist in Asthma

**DOI:** 10.3390/diagnostics13030405

**Published:** 2023-01-22

**Authors:** Sangeetha Vishweswaraiah, Nallur B. Ramachandra, Neha Joshi, Ashwaghosha Parthasarathi, Mohammed Kaleem Ullah, Jayaraj Biligere Siddaiah, Amrutha D. Holla, Samarpana Chakraborty, Anurag Agrawal, Padukudru Anand Mahesh

**Affiliations:** 1 Department of Studies in Genetics and Genomics, University of Mysore, Manasagangotri, Mysore 570006, India; 2 Department of Respiratory Medicine, JSS Medical College, JSS Academy of Higher Education and Research, Mysore 570015, India; 3 Allergy, Asthma and Chest Centre, Krishnamurthypuram, Mysore 570004, India; 4 Rutgers Centre for Pharmacoepidemiology and Treatment Science, New Brunswick, NJ 08901-1293, USA; 5 Centre for Excellence in Molecular Biology and Regenerative Medicine (A DST-FIST Supported Center), Department of Biochemistry (A DST-FIST Supported Department), JSS Medical College, JSS Academy of Higher Education and Research, Mysore 570015, India; 6 Division of Infectious Disease and Vaccinology, School of Public Health, University of California, Berkeley, CA 94720, USA; 7 Center of Excellence, Translational Research in Asthma and Lung Disease, CSIR-Institute of Genomics & Integrative Biology, AcSIR (Academy of Scientific and Innovative Research), Delhi 110025, India

**Keywords:** asthma, *ADAM33*, polymorphism, SNP, ICS, LABA

## Abstract

*ADAM33* has been linked to airway structural changes in patients with asthma, leading to airway hyperresponsiveness, narrowing, and ultimately poor treatment responsiveness. This study aimed to evaluate the genetic association of *ADAM33* SNPs with asthma, disease severity, and treatment responsiveness to ICS+LABA in the South Indian population. In this case–control study (486 controls and 503 cases), we performed genotyping using MassArray for six SNPs of *ADAM33*, namely rs2280091, rs2787094, rs3918396, rs67044, rs2853209, and rs3918392. We studied the association with asthma and treatment responsiveness to ICS+LABA, using genotype, allele frequency distribution, and haplotype analysis. A significant clinical finding of the study was that certain patients in the disease severity group (moderate and mild) showed poor or no improvement after a three-month follow-up of regular ICS+LABA therapy. Of the studied *ADAM33* SNPs, rs2853209 showed an association with asthma. The further analysis of asthma patients according to disease severity suggested an association between moderate disease and the minor allele “T” for rs2853209. The homozygous minor allele of SNP rs2787094 was found to be associated with poorer lung function and the least lung-function improvement after three months of ICS+LABA therapy. The haplotype analysis of six SNPs showed a significant association between the rs2853209 and rs3918396 blocks and asthma. *ADAM33* gene polymorphism has clinical relevance in terms of disease association and response to treatment. SNP rs2853209 seemed most relevant to asthma, and SNP rs2787094 could be a genetic marker for predicting response to ICS+LABA therapy in the study population.

## 1. Introduction

Asthma is a heterogeneous chronic inflammatory disease characterized by reversible airflow limitation [1]. It affects 300 million individuals globally, of which 37.9 million live in India [2,3]. In addition to the already well-established risk factors for asthma, including family, environmental, and individual factors such as smoking and medication adherence, genetics has a significant influence [4,5,6]. Recently, studies such as family and twin association studies [7,8], linkage studies [9], candidate gene studies, and genome-wide association studies [9,10] in asthma have proved strong genetic associations in asthma [8,11]. Structural changes in the airways, or “airway remodeling”, an umbrella term encompassing several modifications in the airways caused by chronic inflammation, is considered one of the significant complications of long-term asthma [12,13,14]. Studies have confirmed that structural changes in the airways impact asthma [15,16]. The first report on genes involved in asthma pathogenesis and airway hyperresponsiveness, other than those concerning non-immune and non-allergic pathways, addressed the positionally cloned asthma and bronchial hyperresponsiveness gene *ADAM33* [17]. This codes for the *ADAM33* protein, a disintegrin and metalloproteinase glycoprotein involved in intercellular and cell–matrix interactions. Soluble *ADAM33* plays a crucial role in airway remodeling [18]. The variations of this gene have been studied in different populations independently and have been found to be associated with asthma [9]. Airway remodeling is involved in airway hyperresponsiveness; airway narrowing; and, finally, poor treatment responsiveness and fixed airflow obstruction. In routine clinical practice, it is difficult to confirm, but the non-reversibility of lung functions after adequate treatment has the potential to be a surrogate marker. Lung functions, especially FEV_1_, may be initially partly reversible and progress to irreversibility in some asthma patients [19].

Medical practitioners have observed that there are variations in response to asthma therapy. This implies that there are three categories of asthma patients in a particular class of asthma therapy (“responders”, “partial responders”, and “non-responders”), and considerations of genetics might aid in the choice of treatment for asthma [20]. In this study, we were keen to find the relationship between *ADAM33* SNPs and asthma, its severity, and the treatment responsiveness after three months of regular inhaled corticosteroids (ICS) and long-acting beta-agonist (LABA) using dosages based on the GINA guidelines for the management of mild, moderate, and persistent asthma [21,22].

## 2. Materials and Methods

### 2.1. Study Population

The study recruited 1074 adult individuals (549 asthma cases and 525 controls, age- and gender-matched). Among them, 503 asthma cases and 486 normal controls were subjected to genotype analysis. The remaining 85 patients did not meet the inclusion criteria (unacceptable spirometry, non-atopic asthma, additional co-morbid conditions). The case and control recruitment period lasted from July 2012 to February 2017. The Institutional Human Ethical Committee, University of Mysore approved the study (IHEC-UOM No.79 Ph.D/2012-13). Written informed consent was obtained from all the asthma patients and participants of the study.

The study did not include individuals under 18 years and asthma patients with other respiratory diseases. Age- and gender-matched controls were selected from the general population in the same geographical area as the asthma cases. The study was conducted on consecutive cases of asthma visiting the department of Respiratory Medicine, JSS Medical College and Hospital, a tertiary-care University teaching hospital. The cases underwent spirometry, satisfying the American Thoracic Society (ATS) standards [23]. Asthma was confirmed according to GINA guidelines for the diagnosis and classification of asthma, i.e., a reversibility of more than 12% and 200 mL on post-bronchodilator spirometry, and asthma severity was classified as mild, moderate, and severe persistent asthma [22]. Spirometry was performed using an Easy-One portable spirometer (NDD Medizintechnik; Zurich, Switzerland). All asthma patients were treatment-naïve (ICS+LABA) at the time of recruitment into the study and used oral bronchodilators as needed before entry into the study. All asthma patients received ICS+LABA for three months, and pre-bronchodilator spirometry was repeated at the end of the three months of the study, when patients were stable and did not have any exacerbations. When there was a viral infection, the treatment was continued, and spirometry was performed at least 2 weeks after the resolution of the viral infection. All asthma patients underwent a skin prick test to confirm atopy; only atopic patients were included in the study. A skin prick test was conducted for common aeroallergens, including weeds, grasses, trees, fungi, house dust mites, and cockroaches. A skin prick test was considered positive when the wheal size was more than 3 mm larger than the saline control. Only patients who were atopic were included in the study. The controls had no asthma symptoms or known family history of asthma and had normal pre-bronchodilator spirometry. The controls underwent only pre-bronchodilator spirometry, as many did not consent to post-bronchodilator spirometry.

### 2.2. Genomic DNA Isolation

The venous blood samples were drawn from the asthma cases and control patients using the venipuncture method. The drawn blood samples were collected in EDTA BD-Vacutainer^®^ PLUS blood collection tubes (Becton Dickinson Ltd., Franklin Lakes, NJ, USA). The Wizard genomic DNA isolation kit of the Promega Company was used to isolate genomic DNA according to the manufacturer’s protocol.

### 2.3. SNP Genotyping

SNP genotyping in the samples was carried out using the Sequenom-MassARRAY platform from Xcelris Genomics Company, Ahmedabad, India. The Sequenom-iPLEX^®^ Gold SNP genotyping platform along with Spectro CHIP was used for the MassARRAY method, and the analysis was performed with MALDI-TOF MS.

### 2.4. Analysis of SNPs of the ADAM33 Gene

This study was performed on six SNPs of *ADAM33* selected based on previous studies and our pilot study [17,24,25,26]. The SNPs and the primers used for SNP genotyping alongside the mass of unextended (E1) and extended (E2) primers in daltons are listed in Table S1.

### 2.5. Statistical Analysis

The clinical parameters of the study population are reported as mean ± SE. The association between variants of *ADAM33* SNPs and asthma was examined using appropriate statistical tests. SPSS software V.19 (SPSS Inc. Chicago, IL, USA) was used to test the association between clinical variables and SNPs. Power analysis was carried out to calculate the power of the study using Quanto software.

The SNP haplotype and disease associations were tested using haploview software (V.4.0) (https://www.broadinstitute.org/haploview/haploview, accessed on 22 November 2017). The software’s case/control option was used to test the associations, and the individuals with >50% missing phenotype were excluded. The >1.0% linked haplotype option was considered to create haplotype blocks. The alluvial chart was generated with Microsoft Excel^®^, and the forest plot was generated using GraphPad Prism software (GraphPad Software Inc., San Diego, CA, USA).

## 3. Results

### 3.1. Demographic and Clinical Profiles of the Study Population

The demographic and clinical profiles of the study patients are summarized in Table 1. Of the asthma patients, 32.8% presented with severe persistent, 42.3% with moderate persistent, and 24.9% with mild persistent asthma (GINA guidelines). The pulmonary function test results for the cases and controls are summarized in Table 2. Asthma patients showed mean FVC, FEV_1_, FEV_1_/FVC%, and PEF (L/s) values lowers than those of the controls. However, lung function within the asthma patients improved after using a bronchodilator.

### 3.2. Improvement in Lung Function in Various Severity Groups upon ICS and LABA Treatment

Asthma patients were treated for three months with inhaled corticosteroids and long-acting beta-agonist. The improvement in their lung function after treatment for three months with ICS+LABA was grouped based on the highest FEV_1_ achieved as normal (FEV_1_ > 80%); partially responsive (some improvement, FEV_1_ 60–80%); or poorly responsive (some improvement, but FEV_1_ still < 60%). We observed that at baseline, 24.9% of the asthma patients had an FEV_1_ > 80, 42.3% between 60 and 80, and 32.8% < 60; after three months of treatment, we observed that 41.6% of the asthma patients had an FEV_1_ > 80, 37.2% between 60 and 80, and 21.2% < 60. The improvements in the asthma patients over the course of three months of treatment are depicted in Figure S1. The duration of asthma (asthma duration < 5 years versus > 5 years) did not seem to influence treatment responsiveness (chi-square 0.18; *p*-value 0.66). A small but important sub-group of patients had worse lung functions than at baseline. A detailed evaluation of the reasons was not performed. Possible reasons include non-adherence, exposure to environmental triggers, or post-viral bronchial hyperresponsiveness.

### 3.3. ADAM33 SNP Distribution in the Study Population

Table S2 summarizes the genotypic and allelic frequency of six SNPs in the asthma patients and controls. The Hardy–Weinberg equilibrium (HWE) was calculated for both cases and controls, showing no deviation in any SNPs except in the control group for rs2280091. The genotype and allele frequency distribution showed a significant association between the TT genotype and asthma for SNP rs2853209 (*p*-value 0.018; odds ratio [95% CI] 0.61 [0.40–0.92]). The minor allele ‘T’ of the same SNP rs2853209 showed a significant association with asthma (*p*-value of 0.035; odds ratio [95% CI] 0.80 [0.65–0.98]), with the minor allele seeming to have a protective effect against asthma (Table S2). The remaining SNPs showed no significant differences between the three asthma severity groups. The table also represents the sample size and odds ratio of the *ADAM33* SNPs. The power analysis for six SNPs showed a relative risk ratio of 1.33 to 1.49 at a power of 80% with a significance of *p* < 0.05.

### 3.4. ADAM33 SNP Association with Disease Severity

We analyzed the genotype and allele frequency distribution of *ADAM33* SNPs among the asthma patients classified as having severe persistent, moderate persistent, and mild persistent cases. We also calculated the odds ratios of the cases (based on severity) vs. the control. The genotype and allele frequency distribution showed an association for SNP rs2853209 between the TT genotype and moderate persistent asthma, with a significant *p*-value of 0.001, as well as the minor allele ‘T’, with a significant *p*-value of 0.002; the minor allele seemed to have a protective effect against moderate persistent asthma but not severe persistent asthma (Table S3).

### 3.5. ADAM33 SNP Association with Lung Function Improvement in Asthma Patients upon ICS and LABA Treatment

We next examined the *ADAM33* SNPs and their association with lung function (FVC and FEV_1_). The SNP rs2787094 (CC) showed significantly lower baseline FVC and FEV_1_ values, with *p*-values of 0.043 and 0.038. The SNP rs3918396 (AA) also showed lower baseline FVC and FEV_1_ values; no significant difference was observed due to the lower number of patients (Table 3 and Table 4). We went on to examine whether the *ADAM33* SNPs were associated with a healthy outcome, in terms of improved lung function and treatment responsiveness, in the asthma patients treated for three months with ICS+LABA. The results are summarized in Table 3 and Table 4. Most of the genotypes showed an improvement in FVC of around 5%, and the FEV_1_ improvement ranged from 4.4% to 10.2%. The SNP rs2787094 (CC genotype) showed a significantly lower improvement in both FVC and FEV_1_, with *p*-values of 0.048 and 0.049. The SNP rs2853209 (TT genotype) showed a significantly lower improvement in FEV_1_ (4.6%) as compared to the AA genotype, which showed a greater improvement in FEV_1_ (10.2%) after three months of ICS+LABA. The SNP rs3918396 (AA genotype) also showed a lower improvement in FVC, but this was not statistically significant due to the fewer patients with that polymorphism.

### 3.6. Haplotype Association of ADAM33 SNPs with Asthma

**Figure 1:**
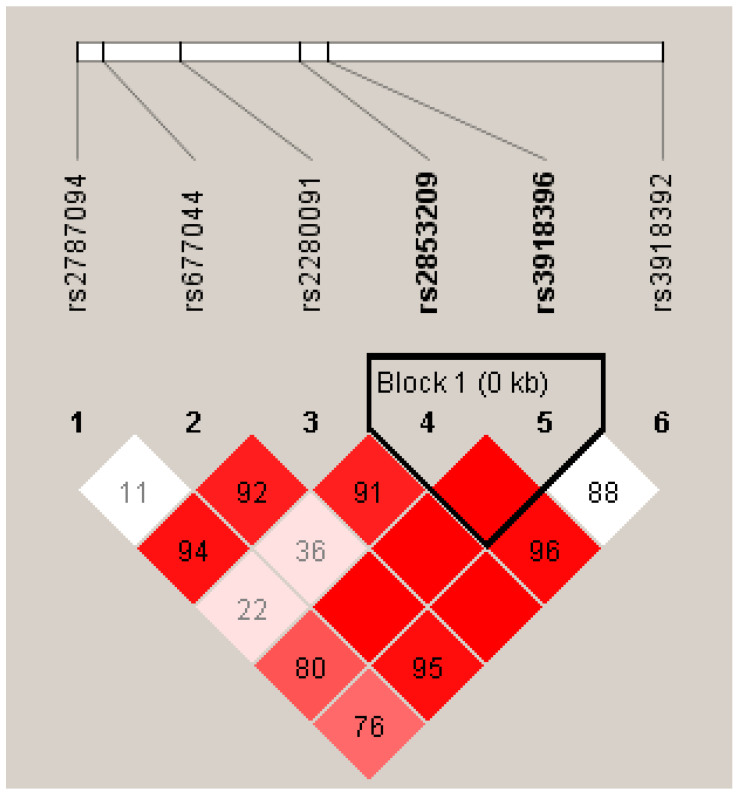
The haplotype block of *ADAM33* SNPs rs2853209 and rs3918396 was significantly associated with asthma. Color gradient description: The red blocks indicate strong linkage disequilibrium and white blocks indicate low linkage disequilibrium.

The association between haplotypes of *ADAM33* SNPs and asthma was obtained through a six-marker haplotype analysis using the haploview tool (Table S4). Of the six markers analyzed, two markers, rs2853209 and rs3918396, which are adjacent to each other, showed a significant association with asthma: haplotype block AG, odds ratio 0.50 (0.46–0.55); haplotype block TG, odds ratio 0.38 (95% CI: 0.33–0.42); and haplotype block AA, odds ratio 0.12 (0.09–0.15). Information on the blocks, haplotypes, and case–control frequencies is presented in Figure 1 and Tables S4 and S5.

## 4. Discussion

*ADAM33*, a metalloproteinase enzyme, plays a significant role in airway structural abnormalities [27], being associated with changes such as epithelial damage, the hyperplasia of smooth muscle cells, fibroblast proliferation, and matrix depositions [28,29]. *ADAM33* has been shown to be associated with these mechanisms [30]. Hence, the aim of this study was to identify the association between *ADAM33* polymorphisms and asthma, disease severity, and treatment responsiveness to ICS+LABA. We observed that the *ADAM33* SNP rs2853209 minor allele (T) was protective against asthma. According to the sub-group analysis, this effect was limited to moderate persistent asthma. The SNP rs2787094 (CC) was associated with significantly worse lung function (FVC and FEV_1_) at baseline as well as poor treatment responsiveness after three months of ICS+LABA. The SNP rs3918396 (AA) was also associated with lower baseline FVC and FEV_1_ values and poor treatment responsiveness, but this relationship was not statistically significant. The TT genotype of the SNP rs2853209 was associated with a significantly greater improvement in FVC and FEV_1_ after ICS+LABA. The GG genotype of the SNP rs3918392 demonstrated an even greater improvement in FEV_1_, but the association was not statistically significant. To the best of our knowledge, this is the first study to investigate the association between *ADAM33* polymorphisms and treatment outcomes after three months of ICS+LABA.

Identifying the genetic variants predisposing an individual to acquire complex diseases is the primary purpose of contemporary human genetics. Case–control studies are the most widely accepted method for examining the association between any risk factor and a condition. However, the critical factor for the success of such case–control studies is achieving an acceptable sample size [31,32]. It has been stated that the frequency distribution of disease-associated alleles is a consequence of the power of the sample size rather than the distribution of disease alleles [33]. A power analysis was performed to estimate the association between SNPs and sample size [34]. All six studied SNPs (rs2280091, rs2787094, rs3918396, rs677044, rs2853209, and rs3918392) showed 80% power with an acceptable statistical significance level of 0.05 and were hence considered for further analysis.

Our study showed that most of the SNPs studied were not associated with asthma at any of the three severity levels (mild, moderate, and severe persistent asthma). One SNP (rs2787094) showed a nearly significant relationship between homozygous alleles and severe persistent asthma, with a *p*-value of 0.093. The same SNP was associated with mild persistent asthma in a North Indian population and a study on the Madeira island population [35,36]. An intronic SNP, rs2853209, showed a highly significant association between the minor homozygous genotype (TT) and asthma (*p*-value 0.001), which was limited to moderate persistent asthma (*p*-value 0.002) and not mild or severe persistent asthma. Though this SNP has been studied in other populations, it did not show any significant association with asthma in other populations [37].

Pharmacogenomics has the potential to help clinicians design personalized treatments for individual asthma patients, which may enhance the effectiveness of treatments. Different asthma patients with a similar disease severity treated with the same medication may respond differently. We observed that the percentage of non-responders or partial responders to treatment among the moderate and severe asthma cases was quite high. After excluding some non-genetic causes, such as adherence, and psychological and environmental factors, one probable reason for this variability is genetic diversity, including SNPs. In recent decades, the use of ICS+LABA has been the gold standard in asthma management, improving health status and quality of life, decreasing exacerbations, and improving lung function [38,39]. However, these therapies have variable outcomes and variable treatment responses, as observed in clinical studies. Few earlier studies have focused on therapy and the response to inhaled ICS+LABA in patients with asthma in relation to different polymorphisms. Only SNPs of the Arg16Gly and *TBX21* genes have been studied to date [40,41]. No previous studies have been conducted on *ADAM33* gene polymorphisms and response to treatment.

Besides playing an important role in health, *ADAM33* also plays an important role in disease. *ADAM33* has been associated with various asthma characteristics, such as bronchial hyperreactivity [42,43], disease progression [44], airway remodeling [28,45], worse lung function [46], and accelerated lung function decline [45]. Consequently, it is possible that the response to therapy is genetically complex [47]. After three months of ICS+LABA treatment, most of the asthma patients with different polymorphisms showed an improvement in FEV_1_ of 4.4% to 10.2%. One of the SNPs, the TT genotype of rs2853209, was associated with significantly higher treatment responsiveness in patients with asthma.

In contrast, the GG genotype of rs3918392 was associated with an even higher improvement in FEV_1_, but this was not statistically significant due to the smaller number of patients. In the case of the SNP rs2787094, a significantly lower (*p*-value 0.0001) improvement in both FVC and FEV_1_ was observed. The rs3918396 SNP (AA genotype) also showed a relatively lower improvement in FVC and FEV_1_, but this was not statistically significant due to the fewer patients with that polymorphism. The association between *ADAM33* SNPs and spirometric parameters indicated the involvement of genetic factors in determining therapeutic response. rs2853209 is an intronic SNP, and rs2787094 is a 3′-UTR variant, wherein the 3′-UTR position affects the mRNA translation regulation [48]. Our study found an association between failure to improve FEV_1_ after a follow-up of three months with ICS+LABA and the SNP rs2787094 minor genotype variant “CC”, which is a probable factor influencing poor treatment responsiveness. This study could not further delineate the exact reasons for the poor treatment responsiveness and *ADAM33* polymorphisms. Possibilities include structural changes in the airway that limited the improvement in FEV_1_ with ICS+LABA. Another possibility is that these patients had small lungs, since *ADAM33* is also important for embryonic lung development, and no further improvement is possible. The Indian population has worse lung function than the Caucasian population, and the possible reasons for this are varied, including antenatal factors, nutrition during childhood, the prevalence of indoor and outdoor air pollution in India, and genetic polymorphisms such as *ADAM33* [49]. Another possibility could be the influence of polymorphisms of the *ADAM33* gene that may lead to the varied expression of *ADAM33*, which may affect smooth muscle function and impact the response to asthma treatment. There is a need for future studies on both longitudinal lung development in children and treatment responsiveness with various alternatives including biologics and *ADAM33* polymorphisms in different ethnic populations across the world.

Every individual’s genotype includes multiple closely linked SNPs, referred to as haplotypes. Each individual’s genome consists of two haplotype alleles, each from one parent [50]. Haplotypes encompass the polymorphisms, variations, and markers on the same chromosome or gene that are inherited together, with a significantly lower or no chance of recombination, since they are located close to each other. Haplotypes are analyzed for two main reasons. Primarily, haplotype alleles may exist in a closer linkage disequilibrium with causal variants compared to single measured SNPs. The other reason is that the haplotypes can be causal variants of significance by themselves [51]. Many computational algorithms are available to measure haplotype frequency and predict haplotype phases from unrelated individuals’ genotype data [52,53,54]. Common haplotype frequencies can be evaluated based on the known marker phenotypes in unrelated individuals of a population [55]. The association between haplotype and asthma in our study was analyzed using haploview software, similarly to other studies [56,57,58]. A study from North India showed a haplotype association between *ADAM33* and asthma, but the SNPs they studied did not include the SNPs rs2853209 and rs3918396 [59] evaluated in this study. In the present study, the rs2853209 and rs3918396 SNPs were associated with asthma. We observed a T-int value of 178.95, with a T-int value greater than 100 denoting that the genomic variants have a tendency to be inherited together. Among the studied SNPs, only these two SNPs were found to be associated significantly with asthma via haplotype analysis. The combination of rs2853209 and rs3918396 has not been observed to be significantly associated with asthma in previous studies. There is a need for the further evaluation of these SNPs and haplotypes in multiple populations and ethnicities to confirm their relevance in the global general population.

**Figure 2:**
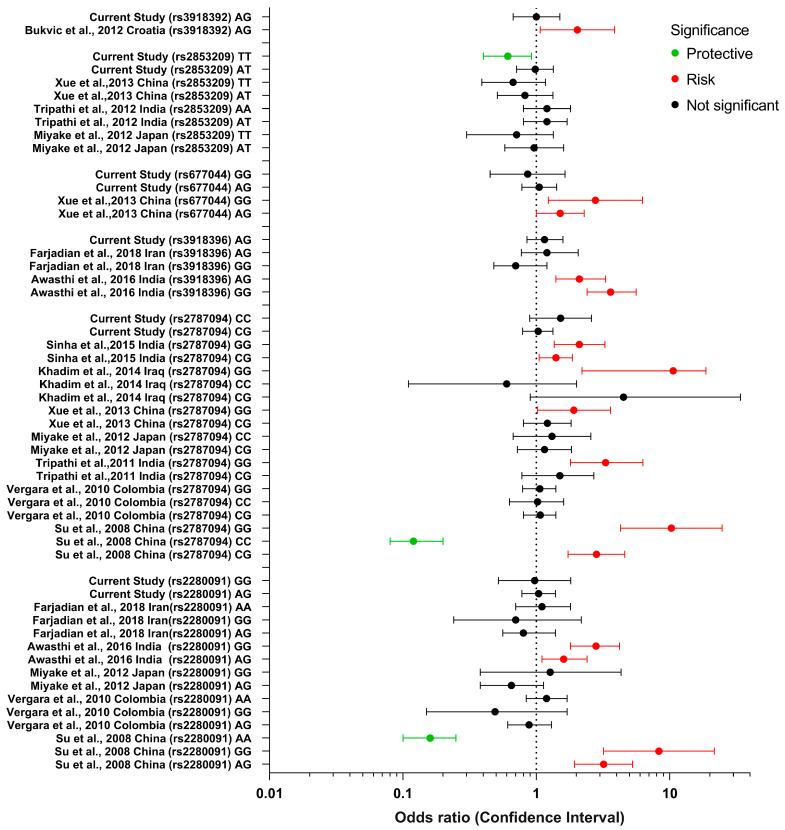
Forest plot of *ADAM33* SNPs genotypes rs2280091, rs2787094, rs3918396, rs677044, rs2853209, rs3918392 and their association with asthma according to various studies conducted globally [56,59,60,61,62,63,64,65,66,67,68].

The review of previous studies on the six SNP polymorphisms of *ADAM33* evaluated in this study is presented in Figure 2. Many of the studies did not observe a significant difference between various *ADAM33* polymorphisms and asthma. Few studies found a strong association between different polymorphisms and asthma. The study of Su et al. [60] (China) observed that patients with the rs2280091 (T1) genotype AG had 3.19 greater odds and those with the genotype GG 8.28 greater odds of suffering from asthma. Awasthi et al. [61] (India) observed a weaker association, with the rs2280091 (T1) genotype AG having 1.6 greater odds and the genotype GG 2.8 greater odds of suffering asthma. For the *ADAM33* SNP rs2787094 (V4), Su et al. [60] (China) observed that patients with the CG and GG genotypes had 2.82 and 10.28 greater odds of suffering asthma. Khadim et al. [62] (Iraq) observed a similarly strong association between the genotypes CG and GG and asthma, presenting 4.5 and 10.6 greater odds, respectively. Sinha et al. [63] (India) observed a weaker association between the CG and GG genotypes and asthma, with 1.40 and 2.10 greater odds, respectively. Xue et al. [64] (China) and Tripathi et al., [59] (India) observed 1.91 and 3.3 higher odds for the GG genotype, but the CG genotype was not significantly associated with asthma. Awasthi et al. [61] (India) observed that patients with the *ADAM33* SNP rs3918396 (S1) genotypes AG and GG had a significant association with asthma, presenting 2.1 and 3.6 greater odds, respectively. For the *ADAM33* SNP rs677044 (V3), Xue et al. [64] (China) observed that patients with the CT and CC genotypes had 1.51 and 2.77 greater odds of suffering from asthma. For the *ADAM33* SNP rs3918392 (F1), Bukvic et al. [65] (Croatia) observed that patients with the GG and GA genotypes had 2.03 greater odds of suffering asthma (Figure 2).

### Strength and Limitations

To our knowledge, this is the first study examining the relationship between *ADAM33* polymorphisms and treatment responsiveness. Our study contained a large number of patients, including both cases and controls. We studied not only asthma but the severity of asthma and treatment responsiveness. The main limitation of this study was that it followed a single-center study design. We did not examine the effects of other ADAM33 polymorphisms that may be relevant for asthma. We were not able to perform post-bronchodilator spirometry on controls. Neither could we perform in vitro functional experiments to assess the potential role of the studied polymorphisms and their influence on treatment responsiveness in asthma patients.

## 5. Conclusions

This study indicated that the *ADAM33* SNP rs2853209 is associated with asthma and treatment responsiveness, with the minor allele conferring improved treatment responsiveness to steroids. Contrastingly, the minor allele of the SNP rs2787094 had poor treatment responsiveness. According to the haplotype analysis, rs2853209 and rs3918396, which are adjacent to each other, showed a significant association with asthma.

## Figures and Tables

**Table 1:** Demographic profile of the study patients.

	Controls (*N* = 486)	Asthma Patients (*N* = 503)
Age		
≤40 years	291 (59.8)	305
>40 years	195	198
Gender		
Male	252	253
Female	234	250
Smoking status		
Smoker	-	57
Non-Smoker	486	446
Family History of Asthma		
Yes	-	213
No	-	290
Asthma Duration		
<1 year	-	61
1–5 years	-	196
>5 years	-	246
Severity according to GINA guidelines		
Mild persistent	-	125 (24.9%)
Moderate persistent	-	212 (42.3%)
Severe persistent	-	166 (32.8%)
Number of Allergens Sensitized		
≤5	-	246
>5	-	257
Wheal diameter		
≤Histamine	-	307
>Histamine	-	196

**Table 2:** Lung function profile of study patients.

Pulmonary Function Test	Controls	Asthma Patients	
	Pre-Bronchodilator	Pre-Bronchodilator	Post-Bronchodilator
FVC (% pred)	88.44 ± 0.511	70.53 ± 0.82	76.02 ± 0.79
FEV_1_ (% pred)	88.84 ± 0.47	66.96 ± 0.89	74.23 ± 0.90
FEV_1_/FVC (% pred)	104.23 ± 0.35	96.69 ± 0.62	102.0 ± 0.66
PEF (L/s)	92.81 ± 0.95	71.60 ± 1.0	77.96 ± 1.05

(FVC: forced vital capacity; FEV_1_: forced expiratory volume in one minute; PEF: peak expiratory flow.)

**Table 3:** Predicted baseline FVC (%) and FVC (%) improvements after three months of inhaled corticosteroids (ICS) and long-acting beta-agonists (LABAs) with various SNPs of *ADAM33* in asthma patients.

			Baseline		Follow-Up (Three Months after ICS+LABAs)	
	Genotype	Asthma Patients	Mean ± SE of Baseline Predicted FVC (%)	95% CI Lower and Upper Limits	Mean ± SE of FVC (%)	95% CI Lower and Upper Limits
rs2280091	AA	353	70.3 ± 0.952	68.4–72.2	75.8 ± 0.916	74.0–77.6
	GA	129	70.0 ± 1.70	66.7–73.4	75.0 ± 1.596	71.8–78.2
	GG	21	77.2 ± 4.78	67.3–87.2	82.0 ± 4.72	72.2–91.8
*p*-value			0.293		0.141	
rs2787094	GG	269	70.5 ± 1.15	68.3–72.8	76.2 ± 1.05	74.1–78.2
	GC	196	71.8 ± 1.28	69.3–74.3	76.8 ± 1.27	74.3–79.3
	CC	38	64.1 ± 2.88	58.2–69.9	68.4 ± 2.92	62.5–74.4
*p*-value			0.043		0.048	
rs3918396	GG	389	70.5 ± 0.945	68.7–72.4	75.8 ± 0.891	74.1–77.6
	GA	107	71.0 ± 1.72	67.6–74.4	76.8 ± 1.75	73.1–79.9
	AA	7	62.4 ± 7.14	45.0–79.9	67.4 ± 7.51	49.1–85.8
*p*-value			0.442		0.222	
rs677044	AA	327	70.4 ± 0.990	68.5–72.4	76.1 ± 0.945	74.1–77.8
	AG	155	70.8 ± 1.58	67.7–73.9	75.7 ± 1.540	72.7–78.7
	GG	21	70.0 ± 4.14	61.4–78.6	75.2 ± 3.349	68.2–82.2
*p*-value			0.913		0.964	
rs2853209	AA	247	71.1 ± 1.06	69.0–73.2	77.1 ± 1.07	75.0–79.2
	AT	195	70.4 ± 1.40	67.7–73.2	74.9 ± 1.31	72.4–77.5
	TT	61	68.5 ± 2.80	62.9–74.1	74.0 ± 2.50	69.0–79.0
*p*-value			0.969		0.374	
rs3918392	AA	435	70.3 ± 0.908	68.5–72.1	75.8 ± 0.853	74.2–77.5
	AG	64	72.0 ± 1.897	68.2–75.8	76.2 ± 2.157	71.9–80.5
	GG	4	72.8 ± 7.962	47.4–98.1	72.5 ± 6.035	53.3–91.7
*p*-value			0.822		0.847	

(FVC—forced vital capacity.)

**Table 4:** Predicted baseline FEV_1_ (%) and FEV_1_ (%) improvements after three months of inhaled corticosteroids (ICS) and long-acting beta-agonists (LABAs) with various SNPs of *ADAM33* in asthma patients.

			Baseline		Follow-Up (Three Months after ICS+LABAs)	
	Genotype	Asthma Patients	Mean ± SE of Baseline Predicted FEV_1_ (%)	95% CI Lower and Upper Limits	Mean ± SE of FEV_1_ (%)	95% CI Lower and Upper Limits
rs2280091	AA	353	66.3 ± 1.061	64.2–68.4	74.2 ± 1.10	72.0–76.3
	GA	129	67.7 ± 1.780	64.1–71.2	73.6 ± 1.62	70.4–76.8
	GG	21	73.6 ± 4.776	63.6–83.5	78.4 ± 4.54	68.9–87.8
*p*-value			0.314		0.459	
rs2787094	GG	269	66.9 ± 1.23	64.5–69.3	74.1 ± 1.16	71.8–76.3
	GC	196	68.5 ± 1.39	65.8–71.3	75.9 ± 1.47	73.0–78.8
	CC	38	59.1 ± 3.48	52.1–66.2	66.4 ± 3.78	58.8–74.1
*p*-value			0.038		0.049	
rs3918396	GG	389	66.8 ± 1.027	64.8–68.8	73.7 ± 0.990	71.7–75.7
	GA	107	67.9 ± 1.890	64.1–71.6	76.0 ± 2.14	71.7–80.2
	AA	7	63.4 ± 8.454	42.7–84.1	73.6 ± 6.37	58.0–89.2
*p*-value			0.746		0.421	
rs677044	AA	327	66.7 ± 1.063	64.6–68.8	74.6 ± 1.10	72.4–76.7
	AG	155	67.4 ± 1.725	64.0–70.8	73.4 ± 1.64	70.2–76.7
	GG	21	67.6 ± 5.187	56.8–78.4	74.2 ± 4.58	64.6–83.7
*p*-value			0.676		0.978	
rs2853209	AA	247	66.5 ± 1.19	64.2–68.9	76.7 ± 1.30	74.1–79.3
	AT	195	68.0 ± 1.48	65.1–70.9	72.4 ± 1.36	69.7–75.0
	TT	61	65.3 ± 3.02	59.3–71.4	69.9 ± 2.62	64.7–75.1
*p*-value			0.722		0.040	
rs3918392	AA	435	66.7 ± 0.975	64.7–68.6	74.0 ± 0.969	72.1–75.9
	AG	64	69.2 ± 2.307	64.5–73.8	75.6 ± 2.37	70.8–80.3
	GG	4	64.3 ± 11.842	26.6–101.9	70.3 ± 11.9	32.4–108.1
*p*-value			0.631		0.826	

(FEV_1_—forced expiratory volume in one minute.)

## Data Availability

All data generated or analyzed during this study are included in this published article and are available from the corresponding author upon reasonable request.

## Supplementary Materials

The following supporting information can be downloaded at: https://www.mdpi.com/article/10.3390/diagnostics13030405/s1, Table S1: Unextended primer (UEP) sequences used for massARRAY analysis along with the extended (E1/E2) alleles and mass in daltons used for the genotyping of *ADAM33* variants in controls and asthma patients, Table S2: Hardy–Weinberg equilibrium (HWE) test, genotype, and minor allele frequency (MAF) of *ADAM33* in healthy controls (C) and asthma patients (P), Table S3: Genotype and allele frequency distribution of *ADAM33* SNPs among controls (C) and asthma patients with severe (Se), moderate (Mo), and mild (Mi) asthma, Table S4: Haplotype analysis of six SNPs of *ADAM33*, Table S5: Details of significant associations between haplotype SNPs of *ADAM33* and controls and asthma patients in the study population, Figure S1: Alluvial chart depicting the changes in FEV_1_ of asthma patients after three months of treatment. FEV_1_: forced expiratory volume in one minute.
