# Supplementary material for: Association between ADAM33 Single-Nucleotide Polymorphisms and Treatment Response to Inhaled Corticosteroids and a Long-Acting Beta-Agonist in Asthma
Source: Diagnostics (Basel). 2023 Jan 22;13(3):405. doi: 10.3390/diagnostics13030405 (PMC9914414; doi:10.3390/diagnostics13030405)
Supplement: Supplementary file 1 [file diagnostics-13-00405-s001.zip › diagnostics-2099881-supplementary.pdf]

diagnostics

MDPI

Article

Association Between *ADAM33* Single-Nucleotide Polymorphisms and Treatment Response to Inhaled Corticosteroids and a Long-Acting Beta-Agonist in Asthma: Supplementary Material

Sangeetha Vishweswaraiah [1], Nallur B. Ramachandra [1], Neha Joshi [2], Ashwaghosha Parthasarathi [3,4], Mohammed Kaleem Ullah [5,6], Jayaraj Biligere Siddaiah [2], Amrutha D. Holla [3], Samarpana Chakraborty [7], Anurag Agrawal [7] and Padukudru Anand Mahesh [2,*]

[1] Department of Studies in Genetics and Genomics, University of Mysore, Manasagangotri, Mysore 570006, India; sangeetha.vsh@gmail.com (S.V.); ramachandra@genetics.uni-mysore.ac.in (N.B.R.)
[2] Department of Respiratory Medicine, JSS Medical College, JSS Academy of Higher Education and Research, Mysore 570015, India; joshi.neha2605@gmail.com (N.J.); bsjayaraj@jssuni.edu.in (J.B.S.)
[3] Allergy, Asthma and Chest Centre, Krishnamurthypuram, Mysore 570004, India; ap2320@rwjms.rutgers.edu (A.P.); amruthadh@yahoo.com (A.D.H.)
[4] Rutgers Centre for Pharmacoepidemiology and Treatment Science, New Brunswick, NJ 08901-1293, USA
[5] Centre for Excellence in Molecular Biology and Regenerative Medicine (A DST-FIST Supported Center), Department of Biochemistry (A DST-FIST Supported Department), JSS Medical College, JSS Academy of Higher Education and Research, Mysore 570015, India; ka7eem@jssuni.edu.in
[6] Division of Infectious Disease and Vaccinology, School of Public Health, University of California, Berkeley, CA 94720, USA
[7] Center of Excellence, Translational Research in Asthma and Lung Disease, CSIR-Institute of Genomics & Integrative Biology, AcSIR (Academy of Scientific and Innovative Research), Delhi 110025, India; samarpana.chakraborty@gmail.com (S.C.); anurag.agrawal@ashoka.edu.in (A.A.)
* Correspondence: pamahesh@jssuni.edu.in; Tel.: +91-9448044003

Table S1. Unextended primer (UEP) sequences used for massARRAY analysis along with the extended (E1/E2) alleles and mass in Daltons used for genotyping of *ADAM33* variants in controls and asthma patients.

Sl No.	SNP rs ID	UEP Sequence	Allele (E1/E2)	Mass of UEP/E1/E2
1	rs2280091	GGGCGGCGTTCACCCCA	G/A	5172.4/5419.5/5499.5
2	rs2787094	GTCCACACTCCCCTG	G/C	4448.9/4696.1/4736.1
3	rs3918396	GGCCATGCTCCTCAGC	A/G	5107.3/5378.5/5394.5
4	rs677044	CCAGCCCTCAGGAACTTCTA	G/A	6021.9/6293.1/6309.1
5	rs2853209	TGGCCTCCCAGTCAAGCG	T/A	5764.7/6036/6091.8

6	rs3918392	GCTCACCTGGAAAGGA	A/G	6176/6447.2/6463.2

Table S2. Hardy-Weinberg Equilibrium (HWE) test, genotype and Minor Allele Frequency (MAF) of *ADAM33* in healthy controls (C) and asthma patients (P).

SNP	Sample Size		Minor allele frequency		Genotype	C	P	OR (95 % CI)	p-value	HWE p-value	
	C	P	C	P						C	P
**rs2280091**	486	503	G-0.16	G-0.17	AA	344	353	Reference		0.017	0.041
					GA	121	129	1.04(0.78-1.39)	0.795		
					GG	21	21	0.97(0.52-1.81)	0.935		
					A	809	835	Reference			
					G	163	171	1.02(0.80-1.28)	0.891		
**rs2787094**	486	503	C-0.24	C-0.27	GG	270	269	Reference		0.235	0.781
					GC	191	196	1.03(0.79-1.33)	0.824		
					CC	25	38	1.52(0.89-2.59)	0.119		
					G	731	734	Reference			
					C	241	272	1.12(0.92-1.37)	0.255		
**rs3918396**	486	503	A-0.10	A-0.12	GG	387	389	Reference		0.568	0.907
					GA	92	107	1.15(0.85-1.58)	0.359		
					AA	7	7	0.99(0.34-2.86)	0.992		
					G	866	885	Reference			
					A	106	121	1.12(0.84-1.47)	0.433		
**rs677044**	486	503	G-0.23	G-0.23	AA	345	327	Reference		0.726	0.588
					AG	121	155	1.05(0.78-1.42)	0.71		
					GG	20	21	0.86(0.45-1.64)	0.66		
					A	811	809	Reference			
					G	161	197	0.99(0.78-1.26)	0.978		
**rs2853209**	486	503	T-0.42	T-0.37	AA	265	247	Reference		0.017	0.761
					TA	147	195	0.98(0.71-1.34)	0.915		
					TT	74	61	0.61(0.40-0.92)	0.018		
					A	677	689	Reference			
					T	295	317	0.80(0.65-0.98)	0.035		
**rs3918392**	486	503	G-0.08	G-0.08	AA	432	435	Reference		0.84	0.562
					AG	52	64	1.0(0.67-1.50)	0.967		

GG	2	4	1.63(0.29-9.0)	0.57
A	916	934	Reference	
G	56	72	1.05(0.73-1.51)	0.783

Table S3. Genotype and allele frequency distribution of *ADAM33* SNPs among controls (C) and patients with Severe (Se), Moderate (Mo) and Mild (Mi) asthma patients.

SNP	Genotype	C	Patients allele count			OR (95 % CI)			p-value		
			Se	Mo	Mi	Se/C	Mo/C	Mi/C	Se/C	Mo/C	Mi/C
**rs2280091**	AA	344	95	153	105	1(Reference)	1(Reference)	1(Reference)	_	_	_
	GA	121	37	51	41	1.11(0.71-1.71)	0.95(0.64-1.38)	1.11(0.73-1.68)	0.644	0.781	0.622
	GG	21	4	7	10	0.69(0.23-2.05)	0.75(0.31-1.8)	1.56(0.71-3.41)	0.505	0.518	0.266
	A	809	227	357	251	1(Reference)	1(Reference)	1(Reference)	_	_	_
	G	163	45	65	61	0.98(0.68-1.41)	0.90(0.66-1.23)	1.21(0.87-1.67)	0.93	0.526	0.26
**rs2787094**	GG	270	76	110	83	1(Reference)	1(Reference)	1(Reference)	_	_	_
	GC	191	47	85	64	0.87(0.58-1.31)	1.09(0.78-1.53)	1.09(0.74-1.59)	0.518	0.609	0.65
	CC	25	13	16	9	1.84(0.90-3.78)	1.57(0.81-3.06)	1.17(0.52-2.61)	0.093	0.183	0.7
	G	731	199	305	230	1(Reference)	1(Reference)	1(Reference)	_	_	_
	C	241	73	117	82	1.11(0.82-1.51)	1.16(0.9-1.50)	1.08(0.80-1.44)	0.492	0.25	0.6
**rs3918396**	GG	387	104	168	117	1(Reference)	1(Reference)	1(Reference)	_	_	_
	AG	92	29	40	38	1.17(0.73-1.87)	1.0(0.66-1.51)	1.37(0.89-2.10)	0.506	0.994	0.156
	AA	7	3	3	1	1.59(0.41-6.27)	0.98(0.25-3.86)	0.47(0.05-3.89)	0.504	0.985	0.485
	G	866	237	376	272	1(Reference)	1(Reference)	1(Reference)	_	_	_
	A	106	35	46	40	1.21(0.80-1.81)	0.99(0.69-1.44)	1.20(0.81-1.77)	0.367	0.997	0.354
**rs677044**	AA	345	92	140	95	1(Reference)	1(Reference)	1(Reference)	_	_	_
	AG	121	38	63	54	0.85(0.54-1.33)	1.01(0.68-1.48)	1.37(0.89-2.09)	0.479	0.973	0.143
	GG	20	6	8	7	0.81(0.31-2.09)	0.77(0.33-1.82)	1.08(0.44-2.65)	0.67	0.554	0.873
	A	811	222	3433	244	1(Reference)	1(Reference)	1(Reference)	_	_	_
	G	161	50	79	68	1.08(0.44-2.66)	0.95(0.69-1.29)	1.19(0.86-1.66)	0.873	0.73	0.285
**rs2853209**	AA	265	63	112	72	1(Reference)	1(Reference)	1(Reference)	_	_	_
	TA	147	53	82	60	0.96(0.61-1.53)	0.89(0.60-1.32)	1.17(0.74-1.85)	0.877	0.564	0.508
	TT	74	20	17	24	0.72(0.39-1.32)	0.37(0.20-0.67)	0.93(0.52-1.65)	0.288	0.001	0.799
	A	677	179	306	204	1(Reference)	1(Reference)	1(Reference)	_	_	_
	T	295	93	116	108	0.85(0.63-1.15)	0.65(0.50-0.86)	0.98(0.74-1.32)	0.305	0.002	0.921
**rs3918392**	AA	432	122	179	134	1(Reference)	1(Reference)	1(Reference)	_	_	_
	AG	52	13	31	20	0.69(0.36-1.32)	1.20(0.74-1.96)	1.06(0.61-1.86)	0.262	0.462	0.833
	GG	2	1	1	2	1.38(0.12-15.38)	1.01(0.09-11.19)	2.76(0.38-19.86)	0.793	0.995	0.313
	A	916	257	389	288	1(Reference)	1(Reference)	1(Reference)	_	_	_

G	56	15	33	24	0.76(0.42-1.37)	1.17(0.74-1.83)	1.18(0.71-1.94)	0.359	0.5	0.523

Table S4. Haplotype analysis of six SNPs of *ADAM33*.

Locus 1	Locus 2	D'	LOD	r^2	CI-low	CI-hi	Dist	T-int
rs2787094	rs677044	0.115	1.92	0.011	0.04	0.19	270	90.93
rs2787094	rs2280091	0.944	17.26	0.066	0.82	0.99	1073	-
rs2787094	rs2853209	0.223	2.08	0.012	0.09	0.34	2311	-
rs2787094	rs3918396	0.808	43.53	0.239	0.73	0.87	2604	-
rs2787094	rs3918392	0.763	26.14	0.147	0.66	0.84	6058	-
rs677044	rs2280091	0.923	12.14	0.053	0.76	0.98	803	170.85
rs677044	rs2853209	0.366	10.96	0.061	0.27	0.45	2041	-
rs677044	rs3918396	1	8.07	0.04	0.77	1	2334	-
rs677044	rs3918392	0.952	50.67	0.271	0.88	0.99	5788	-
rs2280091	rs2853209	0.919	60.88	0.26	0.86	0.96	1238	215.41
rs2280091	rs3918396	1	7.35	0.027	0.77	1	1531	-
rs2280091	rs3918392	1	5.73	0.019	0.72	1	4985	-
rs2853209	rs3918396	1	23.15	0.088	0.92	1	293	178.95
rs2853209	rs3918392	0.964	14.31	0.056	0.83	1	3747	-
rs3918396	rs3918392	0.882	1.24	0.009	0.22	0.99	3454	98.09

[D prime (D′) between two loci, Log of likelihood odds ratio (LOD), Correlation coefficient between two loci (r^2), 95% confidence interval lower and upper bound on D′ (CI-low and CO-hi), Distance between two loci (Dist) and statistic used by HapMap project in haploview software to measure the completeness of information represented by a set of markers in a region (T-int) are represented]

Table S5. Details of significantly associated haplotype SNPs of *ADAM33* among controls and asthma patients under study population.

SNPs	Block	Haplotype	Case-Control frequency	Chi Square	Odds ratio	95% Confidence Interval
**rs2853209, rs3918396**	AG	0.484	0.505, 0.458	3.337	0.50	0.46-0.55
	TG	0.399	0.376, 0.429	4.469	0.38	0.33-0.42
	AA	0.117	0.120, 0.113	0.146	0.12	0.09-0.15

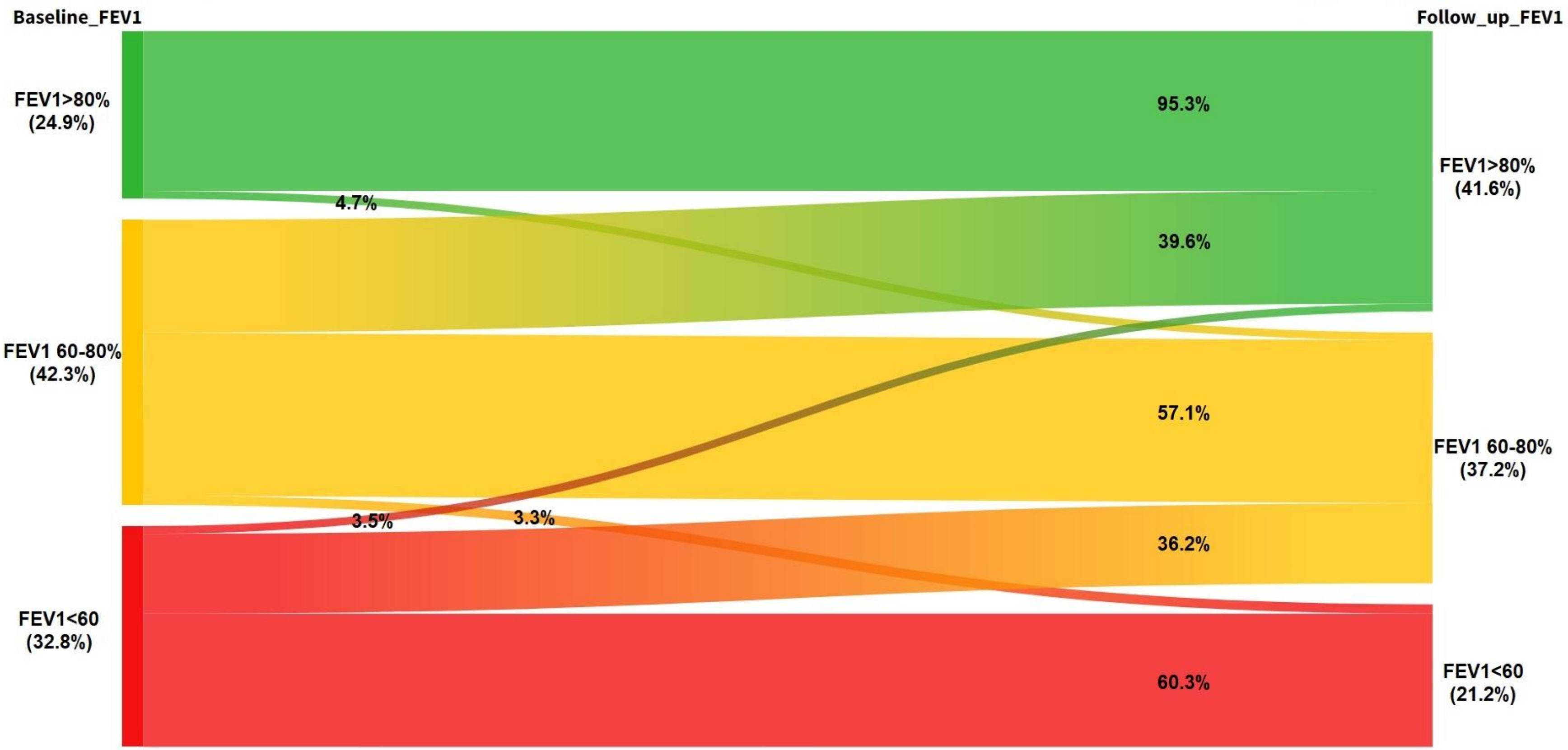

Figure S1. Alluvial chart depicting the changes in FEV_1 of asthma patients after three months of treatment. FEV_1: Forced expiratory volume in one minute.
